# Developing Clinical Prediction Models Using Primary Care Electronic Health Record Data: The Impact of Data Preparation Choices on Model Performance

**DOI:** 10.3389/fepid.2022.871630

**Published:** 2022-06-02

**Authors:** Hendrikus J. A. van Os, Jos P. Kanning, Marieke J. H. Wermer, Niels H. Chavannes, Mattijs E. Numans, Ynte M. Ruigrok, Erik W. van Zwet, Hein Putter, Ewout W. Steyerberg, Rolf H. H. Groenwold

**Affiliations:** ^1^Department of Neurology, Leiden University Medical Hospital, Leiden, Netherlands; ^2^National eHealth Living Lab, Leiden University Medical Hospital, Leiden, Netherlands; ^3^Department of Public Health & Primary Care, Leiden University Medical Hospital, Leiden, Netherlands; ^4^Department of Neurology, University Medical Center Utrecht, Utrecht, Netherlands; ^5^Department of Biomedical Data Sciences, Leiden University Medical Hospital, Leiden, Netherlands; ^6^Department of Clinical Epidemiology, Leiden University Medical Hospital, Leiden, Netherlands

**Keywords:** prediction model, data preparation, electronic health records (EHRs), model performance, model transportability, clinical prediction model

## Abstract

**Objective:**

To quantify prediction model performance in relation to data preparation choices when using electronic health records (EHR).

**Study Design and Setting:**

Cox proportional hazards models were developed for predicting the first-ever main adverse cardiovascular events using Dutch primary care EHR data. The reference model was based on a 1-year run-in period, cardiovascular events were defined based on both EHR diagnosis and medication codes, and missing values were multiply imputed. We compared data preparation choices based on (i) length of the run-in period (2- or 3-year run-in); (ii) outcome definition (EHR diagnosis codes or medication codes only); and (iii) methods addressing missing values (mean imputation or complete case analysis) by making variations on the derivation set and testing their impact in a validation set.

**Results:**

We included 89,491 patients in whom 6,736 first-ever main adverse cardiovascular events occurred during a median follow-up of 8 years. Outcome definition based only on diagnosis codes led to a systematic underestimation of risk (calibration curve intercept: 0.84; 95% CI: 0.83–0.84), while complete case analysis led to overestimation (calibration curve intercept: −0.52; 95% CI: −0.53 to −0.51). Differences in the length of the run-in period showed no relevant impact on calibration and discrimination.

**Conclusion:**

Data preparation choices regarding outcome definition or methods to address missing values can have a substantial impact on the calibration of predictions, hampering reliable clinical decision support. This study further illustrates the urgency of transparent reporting of modeling choices in an EHR data setting.

## Introduction

Electronic health records (EHRs) enable the improvement of quality of care by providing structured information stored in a digital format, straightforwardly derived from routine health care (1, 2). Besides advantages related to the clinical workflow, increased standardization and the pooling of EHR data led to very large datasets that can be of great value for the development of clinical prediction models. EHR-based datasets can reach an unprecedented scale and variety of recorded data, which is practically impossible to achieve in traditional cohort research (3, 4). However, EHRs are designed to record data that are routinely collected during the clinical workflow under a time constraint, in contrast to dedicated prospective cohort studies in which data are collected by trained personnel in a highly standardized manner (5). Consequently, numerous data quality problems are relatively more pronounced in EHR data (6). Previous studies have already enumerated the challenges that the EHR data quality limitations pose for the development of valid clinical prediction models. To overcome these challenges, in many cases, the researcher is faced with difficult or seemingly arbitrary choices in data preparation, for example, regarding the handling of missing predictor values (6–8). Consequently, it may occur in research practice that different data preparation choices will be made for model derivation (or validation) compared with the context of model deployment, which may impact the predictive performance of the model when deployed in clinical practice. The quantification of such choices has not received much attention. In this study, we aimed to evaluate the impact of three previously identified data preparation challenges for EHR-derived prediction models: (i) using a run-in period to define predictors at time zero, (ii) outcome definition, and (iii) methods used to address missing values (6–8). As a case study, we focussed on the estimation of cardiovascular risk in Dutch primary care EHR data.

## Methods

### Data Source

Patient information was derived from general practitioner (GP) practice centers affiliated with the Extramural LUMC Academic Network (ELAN), Leiden, the Netherlands. From the ELAN data warehouse, we defined an open cohort of patients enlisted with ELAN GP practice center from the period of 1 January 2007 to and including 31 December 2018. Patient data included anonymized prescribed medication coded according to the Anatomical Therapeutic Chemical (ATC) classification, laboratory test results performed in primary care, and symptoms and diagnoses coded according to the WHO–FIC recognized International Classification of Primary Care (ICPC) (9, 10). For many GP practice centers, the EHR data on ATC and laboratory test result data became available shortly before or after 2007. Inclusion criteria were age between 40 and 65 years and the absence of a history of cardiovascular disease at cohort entry at the end of the run-in period (see Section “Defining Predictors at Time Zero and a Run-in Period” for details on the run-in period).

### Study Design

**Figure 1:**
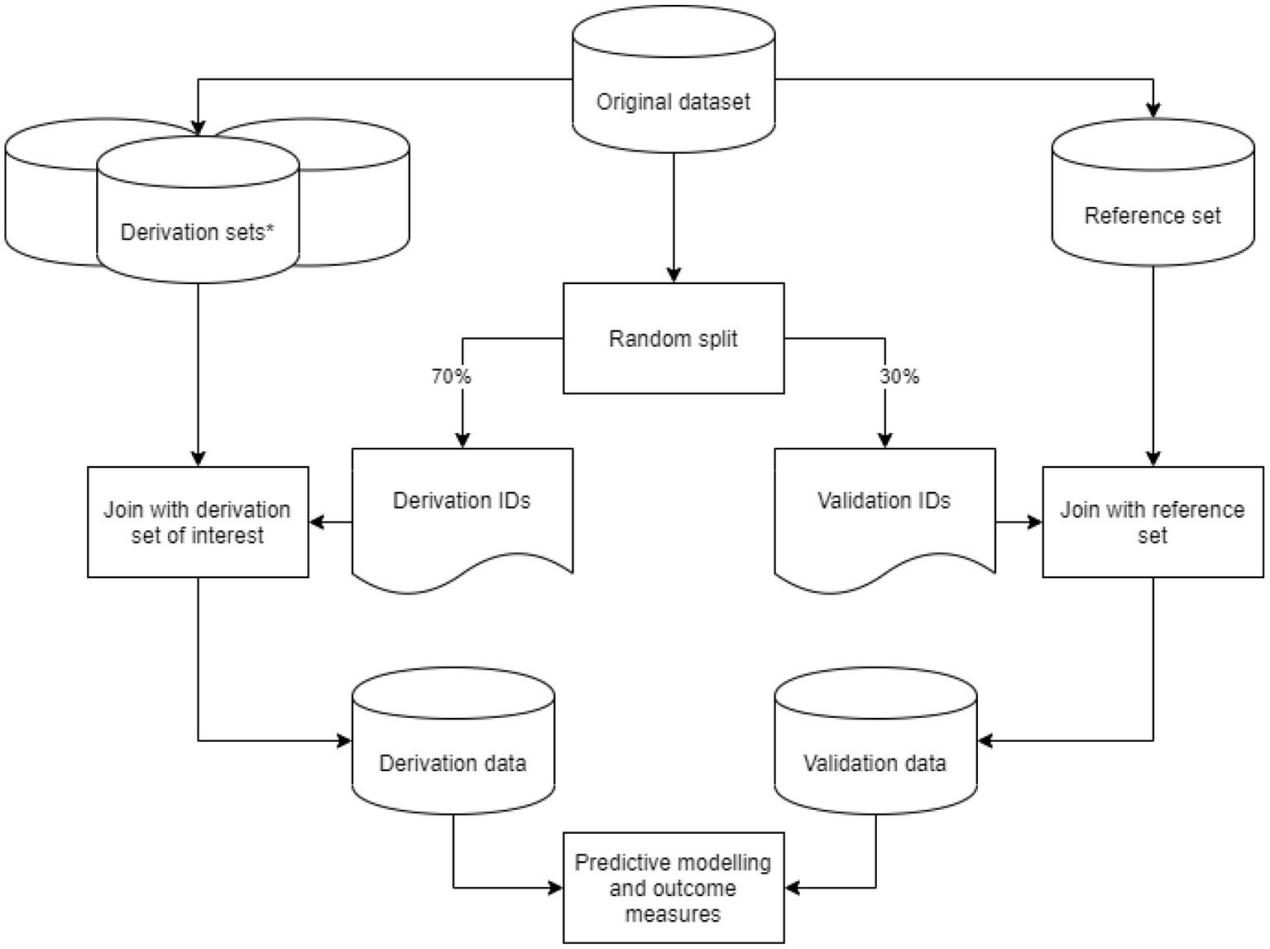
Graphic display of the study design. Graphic display of the study design. *Derivation sets (nine in total: one reference and eight variations) were derived from our original data set, with data preparation steps based on the predefined data preparation challenges.

From our original dataset, we derived nine datasets based on the predefined data preparation challenges. We considered the dataset with a 1-year run-in period, an outcome defined as either ICPC or ATC code, for first-ever main adverse cardiovascular event and multiple imputation as a method for addressing missing values as the reference dataset. In addition to the reference set, we created two derivation sets with a variation in run-in time, four with varying outcome definitions, and two with different methods to address missing values. These eight variations on the reference dataset are described in more detail in the sections below. For each derived dataset, we took a random 70%−30% sample from the original dataset IDs to generate a list of derivation- and validation IDs. Derivation IDs were joined with the derived dataset of interest to generate a derivation set. Validation IDs were joined with the reference set to generate a validation set. Through this approach, we ensured that no individual ID could be in both the derivation and validation sets. We subsequently performed data preparation steps on the derivation and validation sets, fitted the predictive model, and recorded outcome measures. This process was repeated 50 times per derived dataset in a bootstrap procedure for a robust estimate of outcome measures. The study design is graphically displayed in Figure 1.

### Model Development

A multivariable Cox proportional hazards model was developed for predicting the first-ever main adverse cardiovascular events. The following predictors were selected based on prior knowledge: age, sex, mean systolic blood pressure, mean total cholesterol, and smoking as predictors, conforming to the European SCORE model for the prediction of cardiovascular mortality (11).

### Data Preparation Challenges at the Model Development

#### Defining Predictors at Time Zero and a Run-in Period

Time zero (or *t*_0_) is usually defined as the time of enrolment or baseline assessment of covariates. The start of the recording of data in EHRs is in principle the first contact with the healthcare system, which for an individual could be birth or in the prenatal or preconception period. However, as many countries do not have a single, national EHR, health data may be fragmented across EHRs of different healthcare providers, resulting in left-truncation within an EHR database. Hence, there generally is not one clear baseline assessment of predictors. When the time of EHR entry is chosen as *t*_0_, usually no values for laboratory or vital parameter predictors are available. This initial absence of recorded data is in computer sciences also known as the “cold start” problem (12). A possible solution is to define a run-in period, in which all data routinely acquired during a predefined time interval are aggregated into summary variables at the end of this time interval (13). Due to left truncation in our EHR dataset, we chose the start date of our data window as 1 January 2007. We then defined a run-in period of 1 year, meaning that the *t*_0_ was defined as 1 year after the first moment a patient entered the database on 1 January 2007. Additional requirements were age between 40 and 65 years old at *t*_0_. Follow-up ran until the end of the data window on 31 Dec 2018, or until unregistering with an ELAN GP practice center, death, or first-ever main adverse cardiovascular event, whichever came first. Baseline predictors were assessed based on predictor values up until the end of the run-in period. If within this period multiple measurements of systolic blood pressure or total cholesterol were present, the mean value was taken as the baseline measurement. As derivation set variations, we defined run-in periods of 2 and 3 years (see **Table 3**). The reason we chose the 1-year run-in period as a reference was to maximize follow-up time. We chose the mean value as the aggregation method for multiple measurements during run-in, as within this 1-year period measurement values were relatively recent with respect to *t*_0_. Patients who suffered from main adverse cardiovascular events during the run-in period were excluded from analyses.

#### Outcome Definition

Electronic health records are designed to record data that are routinely collected during the clinical workflow. This is different from traditional research, where data are collected by trained personnel in a highly standardized manner (5). This difference could lead to several EHR data quality issues. For instance, a clinical outcome may be present in reality but has not been recorded in the EHR at all or under a different code, possibly leading to the misclassification of outcomes (14). What is more, in an EHR data context one has many more options for outcome definition than in traditional cohort data, such as constructing outcomes using medication or diagnosis codes, or both. Differences in outcome definition in the derivation and target population may cause poor model performance in the target population. The clinical outcome of this study was the 10-year risk of a first-ever major adverse cardiovascular event, and it was based on either event-specific ICPC codes for primary care diagnoses of acute stroke [K90], TIA [K89], acute myocardial infarction [K75], or the start of prescription of event-specific ATC codes for thrombocyte aggregation inhibitors (ticagrelor, picotamide, clopidogrel, dipyridamole, and acetylsalicylic acid). In different derivation sets, the outcome was defined (i) based on ATC codes (without acetylsalicylic acid) or ICPC codes; (ii) based on ATC codes only (including acetylsalicylic acid); (iii) based on ATC codes only, excluding acetylsalicylic acid; or (iv) based on ICPC codes only. The reason for emitting acetylsalicylic acid from the outcome definition is that in the period of our *t*_0_ (2007), it was also prescribed as an analgesic in primary care (15). In addition, Dutch guidelines recommend the prescription of acetylsalicylic acid for stable angina pectoris (16). Consequently, although it may increase the sensitivity for predicting major adverse cardiovascular events, it could come at a cost for specificity. Ticagrelor, picotamide, clopidogrel, and dipyridamole can be regarded as more specific for main adverse cardiovascular events. Although non-cardiovascular mortality could be considered as a competing event, we did not perform a competing risk analysis to limit the complexity of analyses in this study.

#### Missing Values

Since EHR data result from routine care processes, virtually all health data are recorded during clinical contacts for a clinical reason. The missingness of a predictor value is therefore most likely related to the clinical choices of the healthcare professional. In dealing with missing values, it is essential to consider the mechanism of missingness (17). For example, a missing measurement of systolic blood pressure in the EHR, missing completely at random (MCAR), is very unlikely because in clinical practice blood pressure assessment generally requires a medical indication. Missing at random (MAR) will occur if contextual information present in the EHR fully captures the clinician's motives, including those related to the outcome, to assess systolic blood pressure. Arguably, this is unlikely as clinical decision-making takes a large number of biological, psychological, and social factors into account. Missing not at random (MNAR) is therefore the most likely mechanism in this case. In case of MNAR, commonly used imputation strategies such as multiple imputation may result in biased imputed values (18). The combination of an MNAR mechanism with a large extent of missingness in many predictors in EHR data may further increase the risk of biased imputations (19, 20). One way of still leveraging information from the data without requiring sophisticated imputation is the missing indicator method. However, also in this case, similarity of the missingness mechanism between the derivation and target populations is needed (21). Complete case analysis in EHR data could introduce a bias toward the selection of, e.g., sicker patients (22). One should therefore assess how risk of bias resulting from handling missing values may affect the validity of predictions in the target population, and thus the clinical safety of future implementation of the model. Based on this assessment, it may be advisable to discard predictors with a very high extent of missingness and possibly MNAR mechanism altogether.

We imputed the missing continuous predictors of systolic blood pressure and cholesterol using Multivariate Imputation by Chained Equations (MICE). As the input for the MICE algorithm, we used the 30 most important predictors according to a Cox PH model with an elastic net penalty predicting the first-ever cardiovascular events. Although missing values in systolic blood pressure or total cholesterol predictors are unlikely MAR, we multiply imputed because these are important baseline predictors which are used in virtually all cardiovascular risk prediction models. In addition, the aim of this study is not to produce prediction models that can be transported to true clinical settings, but the comparison of different data preparation choices in an EHR data context. Imputations were performed for all derivation and validation sets separately to prevent cross-contamination. We performed multiple visualizations of the complete and completed datasets. Further, we compared the results of the different imputation strategies with the Dutch population means for our age distribution (23). For binary variables, we assumed that the absence of a registration of a clinical entity meant the clinical entity itself was absent. We defined two derivation set variations in which we addressed missing values in the continuous predictors using complete case analysis and mean imputation instead of MICE.

### Assessment of Model Performance at Validation

Models based on the derivation set variations were validated on the reference dataset (see schematic overview in Figure 1). Model performance was assessed *via* the concepts of discrimination (ability of the model to separate individuals who develop the event vs. those who do not) and calibration (the agreement between the estimated and observed number of events). For the evaluation of discrimination, we used the concordance index (c-index), and calibration was assessed using the calibration curve slope and -intercept. For details on these metrics, we refer to the literature (24). We used bootstrap validation with 50 bootstraps for internal validation and simple bootstrap resampling to derive empirical confidence intervals. Analyses were performed using Python version 3.7.

## Results

**Figure 2:**
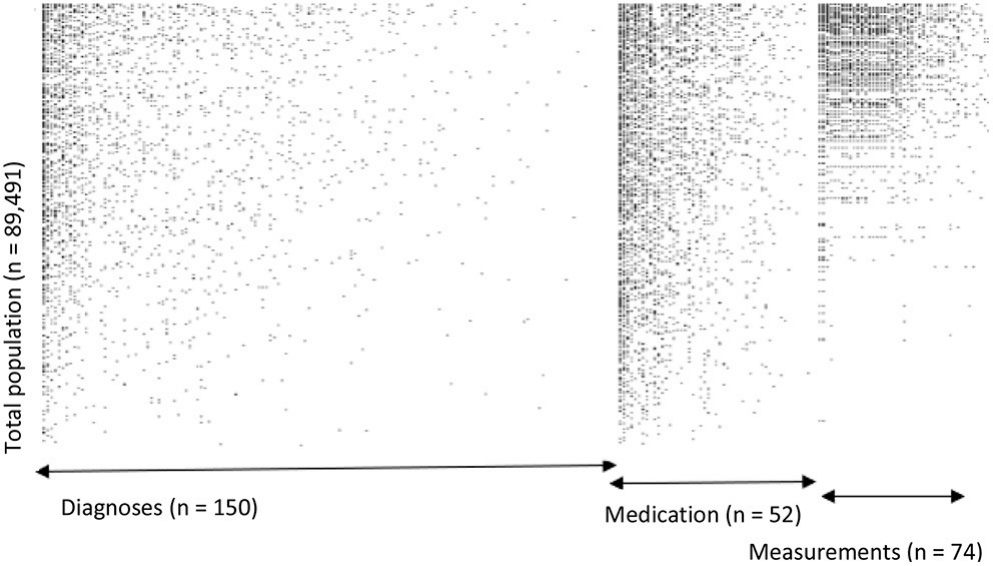
Visualization of data density in Dutch primary care EHR (*n* = 89,491). This figure shows the data density in the EHR for the first year of follow-up of all included patients. The *x*-axis is divided into three different predictor groups: diagnoses (any type of ICPC registration), medications (any type of ATC registration), and laboratory or vital parameter measurements (any type of registration), with each dot representing an EHR registration data point. The *y*-axis represents the entire research population ranked from patients with most data points and descending.

**Figure 3:**
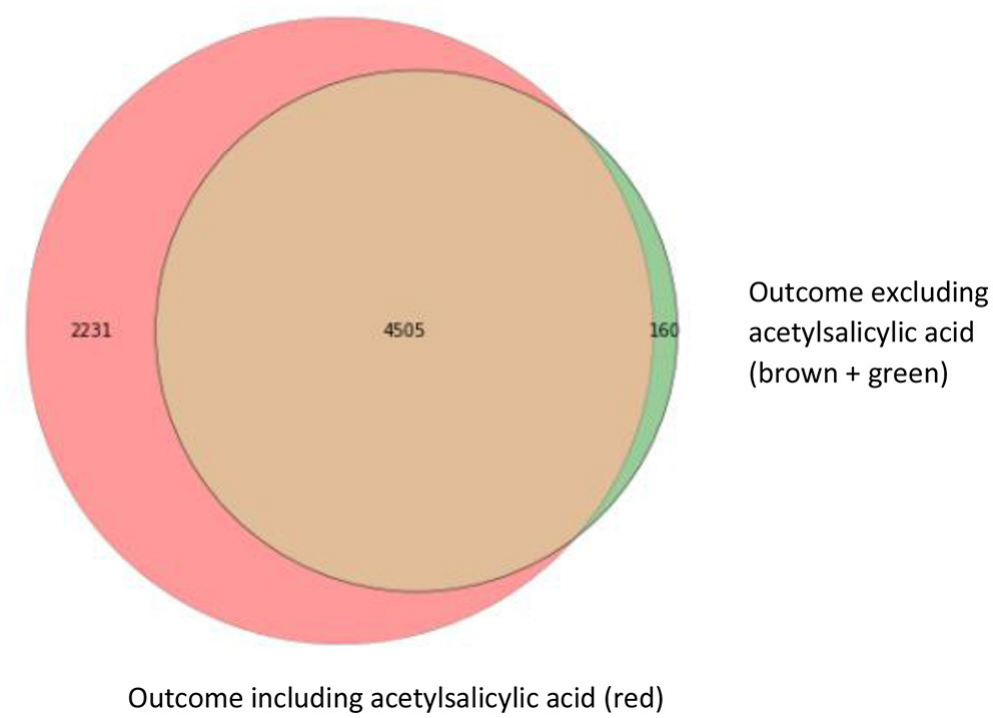
Venn diagram with three different operationalizations for the outcome definition. This Venn diagram shows the numbers of first-ever main adverse cardiovascular event cases resulting from the different outcome definitions: ICPC only (brown; 4,505 cases), ICPC and ATC codes for event-specific medication (clopidogrel, ticagrelor, and dipyridamole) including acetylsalicylic acid (red; 4,505 + 2,231 cases) and ICPC and ATC codes for event-specific medication, excluding acetylsalicylic acid (brown + green; 4,505 + 160 cases).

For our example case study, we included 89,491 patients for analyses in whom 6,736 first-ever cardiovascular events occurred during a median follow-up of 8 years. On an average, patients were 51 years old and 51% were women (Table 1). Visualization of the routine data recorded in the entire population showed that for the majority of patients, of the total of 150 potential diagnoses, no EHR-registrations were present. Although relatively more registrations among the 52 medication and 74 measurement codes were present, for a large part of the population no information was available (Figure 2). For variations in the definition of outcomes, the inclusion of acetylsalicylic acid in the definition resulted in a larger number of cases (Figure 3). Differences were noted between the means in complete cases analysis, imputed by MICE, and the estimated population mean (Table 2).

**Table 1:** Baseline characteristics of participants.

**Baseline characteristics**	**Cases** **(n = 6,736)**	**Controls** **(n = 82,755)**
Age, mean (±SD)	54.8 (6.8)	51.3 (7.3)
Women, *n* (%)	2,849 (42.3)	42,867 (51.8)
Smoking, *n* (%)	494 (7.3)	3,760 (4.5)
**Presence of predictor**
**measurement**, ***n*** **(%)**
Systolic blood pressure	2,302 (34.2)	18,992 (22.9)
Total serum cholesterol	1,637 (24.3)	13,254 (16.0)

**Table 2:** Imputation results of systolic blood pressure and total cholesterol in Dutch primary care EHR data (*n* = 89,491).

	**Systolic blood pressure (mmHg)**	**Total cholesterol (mmol/l)**
Estimated population mean used for mean imputation (SD)	130 (16)	5.7 (1.1)
Sample mean of available measurements/complete case analysis (SD)	136 (17)	5.4 (1.1)
Sample mean after MICE imputation (SD)	132 (10)	5.4 (0.5)

Testing the reference Cox PH model for predicting cardiovascular events on the validation set resulted in a c-statistic of 0.67; 95% CI: 0.67–0.67, a calibration curve intercept of 0.00; 95% CI: −0.01 to 0.00, and -slope of 1.00; 95% CI: 0.99–1.00. Discrimination and calibration were similar for the models based on derivation sets with a 2- or 3-year run-in variations. For the derivation sets with variations in outcome definition, discrimination remained the same but calibration varied greatly, especially when the outcome was based only on ICPC (calibration curve intercept: 0.84; 95% CI: 0.83–0.84, and -slope: 2.31; 95% CI: 2.29–2.32). In this derivation set variation, the event rate was substantially lower compared with the validation set (3.4 vs. 7.5%, respectively), and hence risk was underestimated at the model validation. For models based on derivation set variations in missing data handling, again discrimination was similar to the reference model, but for complete case analysis calibration was substantially worse (calibration curve intercept: −0.52; 95% CI: −0.53 to −0.51, and -slope: 0.60; 95% CI: 0.59–0.60). For this variation also, the total sample size was substantially smaller (around 12% of the reference derivation set) and the event rate was higher (11.4 vs. 7.5% of the validation set), hence risk was overestimated at model validation (Table 3).

**Table 3:** Performance of the models based on derivation set variations compared with the reference model in Dutch primary care EHR data (*n* = 89,491).

		Derivation set characteristics**			**Performance metrics** **		
**Data preparation challenge**	**Derivation set variation description**	**Sample size (range)**	**Percentage events (range)**	**Median follow-up time (days; range)**	**C-statistic (95% CI)**	**Calibration curve intercept (95% CI)**	**Calibration curve slope (95% CI)**
Reference derivation set*	NA	62,644 (62,557–62,730)	7.5 (7.5–7.6)	2,912 (2,904–2,920)	0.67 (0.67–0.67)	0.00 (−0.01 to 0.00)	1.00 (1.00–1.01)
Run-in variations	2 years run-in	58,168 (58,098–58,236)	7.0 (7.0–7.1)	2,832 (2,832–2,832)	0.67 (0.67–0.67)	0.00 (−0.01 to 0.00)	1.00 (0.99–1.00)
	3 years run-in	54,958 (54,884–55,031)	6.4 (6.4–6.5)	2,833 (2,833–2,833)	0.67 (0.67–0.67)	0.02 (0.01 to 0.03)	1.02 (1.01–1.03)
Variations in outcome definition	ATC (excl. ASA) or ICPC	63,376 (63,301–63,448)	5.1 (5.1–5.2)	2,933 (2,925–2,940)	0.67 (0.67–0.67)	−0.40 (−0.41 to −0.40)	0.67 (0.66–0.67)
	ATC only	63,518 (63,436–63,597)	7.5 (7.4–7.5)	2,916 (2,909–2,922)	0.68 (0.68–0.68)	−0.01 (−0.02 to 0.00)	0.99 (0.99–1.00)
	ATC (excl. ASA) only	64,739 (64,662–64,819)	4.6 (4.5–4.6)	2,968 (2,956–2,979)	0.68 (0.68–0.68)	−0.52 (−0.53 to −0.51)	0.59 (0.59–0.60)
	ICPC only	64,089 (63,998–64,180)	3.4 (3.3–3.4)	3,025 (3,010–3,040)	0.66 (0.66–0.66)	−0.84 (−0.85 to −0.83)	0.43 (0.43–0.44)
Missing data method variations	Complete Case	7,601 (7,573–7,629)	11.4 (11.3–11.5)	2,425 (2,409–2,442)	0.62 (0.62–0.62)	0.53 (0.51 to 0.54)	1.69 (1.67–1.71)
	Mean imputation	62,548 (62,478–62,618)	7.5 (7.5–7.6)	2,910 (2,901–2,918)	0.66 (0.66–0.66)	0.01 (0.00 to 0.02)	1.01 (1.00–1.02)

*ASA, acetylsalicylic acid; ICPC, International Classification of Primary Care diagnosis codes; ATC, Anatomical Therapeutic Chemical medication codes.*

*The reference derivation and validation set is defined by 1 year run-in, imputation using MICE, and outcome definition based on ICPC or ATC codes (including aspirin).

**Derivation set characteristics and performance metrics are given as average across 50 bootstrap samples.

## Discussion

This study shows that for the prediction of first-ever cardiovascular event risk using Dutch primary care EHR data, different data preparation choices regarding the outcome definition (first-ever cardiovascular events) and methods used to address missing values in the derivation set can have a substantial impact on model calibration, while model discrimination remains essentially the same. The large changes in calibration curve intercept and -slope could be explained by the changes in the percentage of events that resulted from the different data preparation choices in the derivation set variations. A drop in the proportion of events in the derivation set variations compared with the reference derivation set (e.g., defining outcome using only ICPC codes) led to a decrease in the calibration curve intercept and a rise in the proportion of events (e.g., in case of using complete case analysis to handle missing values) led to an increase. These deteriorations of calibration may be of substantial clinical significance when a prediction model is used in clinical practice, for example within a clinical decision support tool. To evaluate a model on its utility to support clinical decisions, calibration is a more relevant performance metric than model discrimination (24, 25).

Previous research already identified numerous methodological challenges for the development of clinical risk prediction models using EHR data (6–8). To the best of our knowledge, this is the first study that quantifies the impact that different data preparation choices in an EHR data setting have on model performance. The three data preparation challenges that are treated in this study do relate to previous studies that focus on EHR-based data. One study used multiple methods for aggregation of baseline measurements during a run-in period and found that simple aggregations such as the mean are sufficient to improve model performance (26). Further, several studies illustrate the difficulty of choosing an outcome definition in an EHR data context, especially due to the substantial variations of misclassification for different types of EHR diagnosis codes. In one example, the positive predictive value (PPV) of the diagnosis code for chronic sinusitis was 34 vs. 85% for nasal polyps. With the additional information of evaluation by an otorhinolaryngologist, the PPV of the latter rose to 91% (27, 28). One study quantified the effect on the model performance of misclassification in predictors instead of the outcome using the CHA_2_DS_2_-VASc prediction rule as a case study. The substantial misclassification of predictors did not affect overall model performance, but it did affect the risk of the outcome with a certain CHA_2_DS_2_-VASc score (29). In this study, we focussed on the influence of misclassification in the outcome on model performance, but also misclassification in predictors should be taken into account when developing a clinical prediction model using EHR data. Regarding the imputation of EHR predictor values that are likely MNAR, studies found that there may still be options for imputation if missingness structure is explicitly modeled. Methodologies such as Bayesian analysis may be specifically suited for this purpose (6, 30). However, further research into this topic is needed. One option is to discard a variable altogether, especially in case of large extent of missingness (19). In the future, missingness in EHR data might be reduced by more systematic data capture, or through automated analysis of free text using natural language processing techniques (31).

### Strengths and Limitations

Several methodological limitations need to be taken into account to interpret our study results. First, in our EHR data, no reference standard for the definition of the outcome was present, complicating the interpretation of the model results. It should also be noted that for many EHR-derived diagnoses, available reference standards may a certain degree of misclassification (32). Therefore, the researcher needs to work with the routine data that are available, often resulting in difficult or seemingly arbitrary choices regarding outcome definition. In this study, we focused on the relative impact on the model performance of different outcome definitions, instead of a comparison with a reference standard for outcome. We assumed that the definition used in the reference derivation set (ATC including acetylsalicylic acid or ICPC) was most sensitive because of the broad inclusion of thrombocyte aggregation inhibitors that are prescribed after cardiovascular events. However, in the first years of our follow-up period, acetylsalicylic acid was also prescribed in a primary prevention setting, thus the outcome according to ATC excluding acetylsalicylic acid is considered as the most specific.

Second, regarding the different choices in addressing missing data, in the reference derivation set, systolic blood pressure and blood cholesterol were imputed using MICE despite the large extent of missingness in these predictors. As the predominant missingness mechanism is likely MNAR as has been argued in Section Missing Values, these imputation results are likely biased to some extent. The density of data points across all diagnosis, medication, and measurement codes showed that for a large number of patients, the lack of information often extended to the entire dataset, which also hampers reliable imputation. We compared imputation results with expected population means and indeed found a moderate difference. Although these likely biased estimates may not be a problem at internal validation, they may be at external or prospective validation when the missingness mechanism itself is not transportable to these new data environments. Third, although non-cardiovascular mortality could be considered a competing event, we did not perform a competing risk analysis to limit the complexity of analyses in this paper. The number of non-cardiovascular deaths recorded during follow-up was 2,838, which represents only 3% of the total study population. Therefore, the effect of non-cardiovascular mortality as a competing event on potential overestimation of the cumulative incidence of cardiovascular events was likely limited. Finally, the discriminative performance of our models is relatively low. An explanation for the relatively poor discrimination is the limited number of predictors selected for the model and the limited age range of 40–years, based on our conformity with the SCORE model. The discriminative performance found in our study, however, is not uncommon for clinical prediction models used in practice, and is comparable with that of, for e.g., the CHA_2_DS_2_-VASc prediction rule (33). In addition, compared with discrimination, calibration is of more interest to compare model performance because of the future intended use of the models to support clinical decisions (24). Strengths of this study include the very large sample size of our routine care dataset and a large number of derivation set variations (eight) that we used to assess the impact of difficult or seemingly arbitrary choices in data preparation on model performance.

### Future Considerations

Our findings stress the importance of carefully considering differences in data preparation choices between the population used for model derivation compared with the target population for model validation or deployment because these differences may lead to substantial miscalibration. In essence, this study's methodology of including multiple derivation set variations could be seen as a form of sensitivity analysis to assess transportability of the model to a clinical setting in which different data preparation choices are made. However, all data used in this study were derived from the same EHR data source (ELAN). Therefore, we could not formally test transportability across different EHR data sources. Still, this study further illustrates the need for the transparent reporting of choices in model development studies and model calibration in validation studies. This could be done using, e.g., the RECORD statement for reporting on data preparation choices using routinely collected health data in EHR, and the TRIPOD statement for reporting on clinical prediction model development (34, 35).

## Conclusion

Our findings support that for developing clinical prediction models using EHR data, variations in data preparation choices regarding outcome definition and dealing with missing values may have a substantial impact on model calibration, while discrimination remains essentially the same. It is, therefore, important to transparently report data preparation choices in model development studies and model calibration in validation studies.

## Data Availability Statement

The original contributions presented in the study are included in the article, further inquiries can be directed to the corresponding author.

## Ethics Statement

Ethical review and approval was not required for the study on human participants in accordance with the local legislation and institutional requirements. Written informed consent for participation was not required for this study in accordance with the national legislation and the institutional requirements.

## Author Contributions

HO wrote the first draft of the manuscript. HO and JK performed statistical analyses. MN organized the database. NC, EZ, HP, ES, and RG contributed to the conception and design of the study. All authors contributed to manuscript revision, read, and approved the submitted version.

## Funding

HO was funded by a personal Dekker Junior Clinical Scientist Grant (2018T082) and the Innovation Grant (2018T016) from the Dutch Heart Foundation, and a ZonMw Gender & Prevention Grant (555003014). MH was supported by a personal Veni and Vidi grant from ZonMw, a Dekker Junior Staff Member Grant from the Dutch Heart Foundation (2011T055), and a Fellowship Grant from the Dutch Brain Foundation (F2014(1)-22). JK and YR received funding from the European Research Council (ERC) under the European Union's Horizon 2020 Research and Innovation Program (grant agreement No. 852173).

## Conflict of Interest

The authors declare that the research was conducted in the absence of any commercial or financial relationships that could be construed as a potential conflict of interest.

## Publisher's Note

All claims expressed in this article are solely those of the authors and do not necessarily represent those of their affiliated organizations, or those of the publisher, the editors and the reviewers. Any product that may be evaluated in this article, or claim that may be made by its manufacturer, is not guaranteed or endorsed by the publisher.
